# Ultralow noise miniature external cavity semiconductor laser

**DOI:** 10.1038/ncomms8371

**Published:** 2015-06-24

**Authors:** W. Liang, V. S. Ilchenko, D. Eliyahu, A. A. Savchenkov, A. B. Matsko, D. Seidel, L. Maleki

**Affiliations:** 1 OEwaves Inc., 465 North Halstead Street, Suite 140, Pasadena, California 91107, USA

## Abstract

Advanced applications in optical metrology demand improved lasers with high spectral purity, in form factors that are small and insensitive to environmental perturbations. While laboratory-scale lasers with extraordinarily high stability and low noise have been reported, all-integrated chip-scale devices with sub-100 Hz linewidth have not been previously demonstrated. Lasers integrated with optical microresonators as external cavities have the potential for substantial reduction of noise. However, stability and spectral purity improvements of these lasers have only been validated with rack-mounted support equipment, assembled with fibre lasers to marginally improve their noise performance. In this work we report on a realization of a heterogeneously integrated, chip-scale semiconductor laser featuring 30-Hz integral linewidth as well as sub-Hz instantaneous linewidth.

## Abstract (web summary)

Optical metrology applications require lasers with high spectral purity but on-chip devices with sub-100 Hz linewidth are yet to be realized. Here, Liang *et al.* present a heterogeneously integrated, chip-scale semiconductor laser with 30 Hz integral linewidth and sub-Hz instantaneous linewidth.

## Graphical abstract

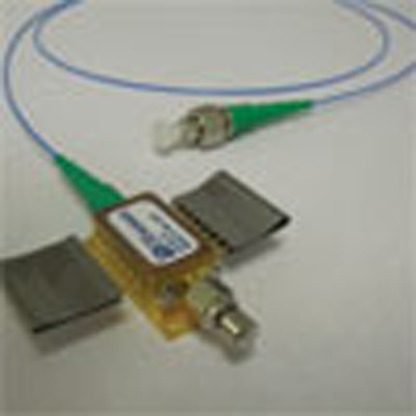

Lasers with high spectral purity^1^ are key elements that determine the ultimate achievable sensitivity in advanced optical frequency metrology^2^,^3^,^4^,^5^,^6^. A number of diverse applications, such as atomic clocks^7^,^8^,^9^,^10^, high-resolution spectroscopy^11^,^12^,^13^, quantum information science^10^,^14^, high-resolution optical sensing, light detection and ranging (LIDAR) and spectrally pure photonic microwave generation^15^,^16^,^17^ require ultranarrow line lasers to optimize their performance. Beyond this, high data rate optical communications, including coherent communications^18^,^19^, require lasers with low frequency and amplitude noise. In most instances, solid state or fibre lasers stabilized to external super cavities (high-finesse ultrastable cavities) are used to fulfil these needs. Because of the size and complexity of super cavities, ultranarrow linewidth lasers are typically bench-top systems suitable for the laboratory environment. There are several types of fieldable super cavities and lasers based on them reported so far^20^,^21^,^22^; however, they are large in size and relatively high in complexity.

Semiconductor lasers have the potential to serve applications where size, weight and power are important operational parameters. Nonetheless, the inherent linewidth of semiconductor lasers, ultimately limited by the small size and quality factor (*Q*) of the laser cavity, is typically in the MHz range, making the use of a super cavity or an atomic transition mandatory for their line narrowing and stabilization^5^,^12^,^23^,^24^,^25^,^26^,^27^,^28^,^29^. This requirement, however, diminishes the benefits of small size and power consumption that are desirable features of semiconductor lasers.

Applications related to spectroscopy of narrow atomic or molecular transitions require ultralow noise lasers at a variety of wavelengths in the ultraviolet, visible, and mid- and far-infrared. Depending on the wavelength in these applications, practical super cavities may be difficult to realize even where semiconductor lasers are available. It is highly desirable to devise an approach where limitations related to mirrors and other elements of super cavities do not compromise the availability of high spectral purity semiconductor lasers. Crystalline whispering gallery mode (WGM) microresonators are mirrorless structures operational at any wavelength within the transparency range of the host material^30^. This makes them suitable for stabilization of any kind of lasers emitting in the ultraviolet, visible and infrared.

In this work we report on the development and study of a chip-scale heterogeneously integrated diode laser stabilized by self-injection locking to a crystalline WGM microresonator. The laser, the associated optics and the resonator itself are all integrated in a single package and occupy less than 1 cm^3^ volume. Both the instantaneous and integral linewidths of the laser (see ref. 31 for a detailed discussion regarding definition of linewidth of a laser) are at least two orders of magnitude smaller than previous demonstrations of resonator-stabilized semiconductor lasers^31^,^32^,^33^,^34^,^35^,^36^. The spectral purity of the laser, represented by its frequency noise, is also higher than values achieved with locking of a fibre laser to a microresonator of similar size, as well as a Brillouin laser based on pumping a microresonator with highly coherent light emitted by a fibre laser^15^,^37^. The superior stability of operation of this laser is achieved without a vacuum vessel or vibration isolation.

## Results

### Linewidth of a diode laser

Spectral purity of a diode laser is fundamentally limited by quantum noise (see, for example, ref. 38)


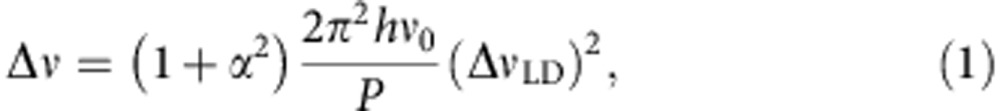


where Δ*ν*_LD_ is an equivalent to full width at half maximum of the cold laser cavity, *α* is the linewidth-broadening factor^39^,^40^, *ν*_0_ is the carrier frequency and *P* is the output power. A conventional distributed feedback (DFB) semiconductor laser has MHz range linewidth. Indeed, if we assume *α*=2.5, *ν*_0_=2 × 10^14 ^Hz, *P*=20 mW and Δ*ν*_LD_=50 GHz we find Δ*ν*=2 MHz. This value is three orders of magnitude larger as compared with the linewidth of a good bench-top fibre laser. An external cavity must be used to improve the diode laser performance, usually at the cost of a significant increase in the device dimensions. This is the motivation behind stabilization of the diode laser with monolithic microresonators.

To realize a low noise, chip-scale stabilized diode laser several contradictory requirements must be considered. For example, the stability of a laser oscillator depends on its geometrical size, unless the oscillator is stabilized with an atomic or molecular transition or self-referenced in a special way. This limitation results from thermodynamics of a laser's energy storage element. For chip-scale applications, these elements are usually nano- and micro-scale resonators. Thermodynamics considerations limit the stability of temperature and dimensions of laser resonators and, consequently, their frequency stability. For instance, Allan deviation of a resonator a few millimetres in size reaches ∼10^−13^ at 1-s integration time, which implies that a semiconductor laser linewidth can be reduced only to tens of Hz by locking the laser to such a resonator. A smaller reference cavity has worse short-term stability. Therefore, highly stable devices must be large enough to support the targeted stability. This is in contradiction to the common desire to reduce the resonator size down to a volume corresponding to the size of the wavelength.

Achieving the fundamental thermodynamic limit requires reducing both the power of light in the resonator, and the absorption of light in the mode volume. Absorption results in power-dependent change of the temperature of the resonator and destabilizes the mode frequency. Attenuation in a microresonator is reduced if the quality factor of the device is limited by coupling to the external environment, and not by the material loss. Thus, it is important to reduce the quality factor of the resonator with respect to the maximal achievable value. This requirement opposes the necessity for a narrow bandwidth reference cavity as a prerequisite to achieving low noise oscillation. Furthermore, reducing the power contradicts the requirement of high signal to noise ratio to achieve effective locking of the laser to the resonator.

A highly transparent material has to be utilized for microresonator fabrication to minimize material absorbtion of the laser light. It has been shown that crystals are the most transparent materials in virtually all regions of the electromagnetic spectrum. For instance, calcium fluoride (CaF_2_) has linear absorption at the level of 3 × 10^−7 ^cm^−1^ at 1,550 nm (ref. 41), which is more than an order of magnitude smaller than the absorption of fused silica and five orders of magnitude smaller than absorption of silicon at this wavelength. Thus, wide gap (dielectric) optical crystals are extremely attractive as the host material for reference microresonators.

Electrical locking schemes are usually implemented to stabilize a diode laser to an optical resonator. In this approach, either the resonator or the laser frequency must be modulated to generate an error signal for feedback to the laser current and/or its temperature. The laser should be compatible for fast enough actuation to achieve efficient feedback. While this is a robust and widely used technique, the feedback circuit frequently generates noise peaks in the frequency spectrum of the locked laser.

### Self-injection locking

Self-injection locking^32^,^33^,^34^ is one of the most efficient techniques for locking a laser to a monolithic resonator. The method is based on resonant Rayleigh scattering in the resonator because of surface and/or volumetric inhomogeneities^42^,^43^. With Rayleigh scattering, some amount of light reflects back into the laser when the frequency of the emitted light coincides with the frequency of a selected resonator mode. This provides a fast optical feedback to the laser, enabling significant reduction in linewidth. In the work reported here we achieved seven orders of magnitude reduction in the laser linewidth with this approach. Since self-injection locking does not require any modulation and electronics, the laser can be tightly packaged, which simplified its thermal stabilization, and also reduces the influence of ambient acoustic noise on the laser frequency. Such a device can also be used as a master laser for pumping high-power lasers for metrology and remote sensing applications.

The efficiency of self-injection locking can be described by an equation derived for the reduction of the close-in (low Fourier frequency) phase noise of the laser^44^,^45^,^46^


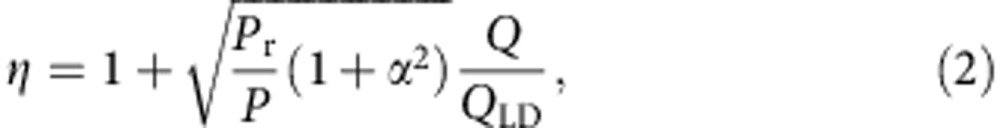


where *P*_r_ is the power reflected back to the laser mode, *Q* and *Q*_LD_ are the quality factors of the reference and laser cavities. The higher the quality factor of the resonator the higher is the noise suppression equation (2). This again points to crystalline resonators as the best choice for laser stabilization, as their Q-factor can exceed 10^11^ (ref. 41). Substituting reasonable numbers in equation (2) for *P*_r_/*P*=0.3, *α*=2.5 and *Q*=1 × 10^9^ we obtain *η*≃4 × 10^5^. It means that the linewidth of a diode laser can be improved by nearly a factor of a million. In our experiment we achieved an even larger reduction, which can be attributed to the unusually strong optical feedback from the microresonator to the laser, which is not described by the conventional perturbation theory of self-injection locking^44^,^45^,^46^. In our case the optical feedback not only stabilizes the frequency but also slightly reduces the threshold of the laser.

### Experiment

To perform the measurements reported here, we built two identical lasers. Significant attention was paid to thermal stabilization of laser packages to reduce the technical noise due to environmental temperature fluctuations^31^. By carefully designing the thermal package we achieved a thermal response time exceeding 50 s. We also fabricated a hermetic package for the reference resonator to increase its environmental stability.

**Figure 1:**
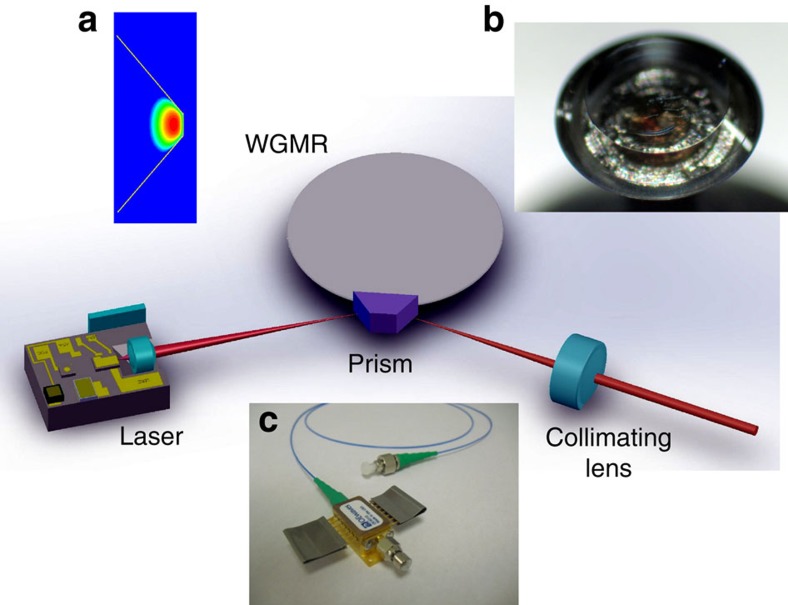
Schematic of the experimental set-up. Light from the pump laser (a semiconductor DFB laser) enters the whispering gallery mode resonator (WGMR) through the prism. Part of light is reflected back to the laser because of Rayleigh scattering in the resonator. The light exiting the prism is collimated and used for processing. Insets show power distribution in the resonator mode (**a**), a picture of the actual resonator (**b**) as well as a picture of the packaged laser (**c**).

The laser set-up is schematically shown in Fig. 1. Light emitted from a 1,550-nm semiconductor DFB laser mounted on a ceramic submount is collimated and sent into a MgF_2_ WGM resonator using a glass coupling prism. The resonator has an unloaded *Q*-factor of ∼6 × 10^9^, and its loaded quality factor is 6 × 10^8^. Surface Rayleigh scattering^43^ results in forming WGM doublets with frequency splitting on the order of 100 kHz.

The power at the output of the laser chip is 20 mW and the power at the exit of the prism is ∼10 mW. The reduction in power occurs because ∼5 mW was lost through a second prism coupler, not depicted in Fig. 1, used to track the field accumulation inside the resonator; and another 5 mW was reflected back to the laser chip, facilitating self-injection locking. A small amount of light was lost because of imperfect mode-matching. It is important to note that the operation of the laser is stable with such a strong feedback, which apparently contradicts the results of earlier theoretical studies^44^. The laser frequency can be pulled by the WGM frequency in a range exceeding 4 GHz. Synchronous feedback to the laser current and temperature allows achieving orders of magnitude broader tunability without any efficiency dips.

To characterize this performance level, we thermally tuned the frequencies of the lasers to be ∼10 GHz apart, merged their beams in space, coupled them into a fibre and combined them at a photodiode using a fibre coupler. The optical power level of the lasers at the photodiode was reduced to 0.25 mW each because of loss in a diagnostic optical train that includes a beam splitter, and an isolator, in addition to the insertion loss in the fibre. The radio frequency (RF) beat note signal at the output of the photodiode was amplified and evaluated using a commercial phase noise measurement system (OEwaves' Automated PNTS).

**Figure 2:**
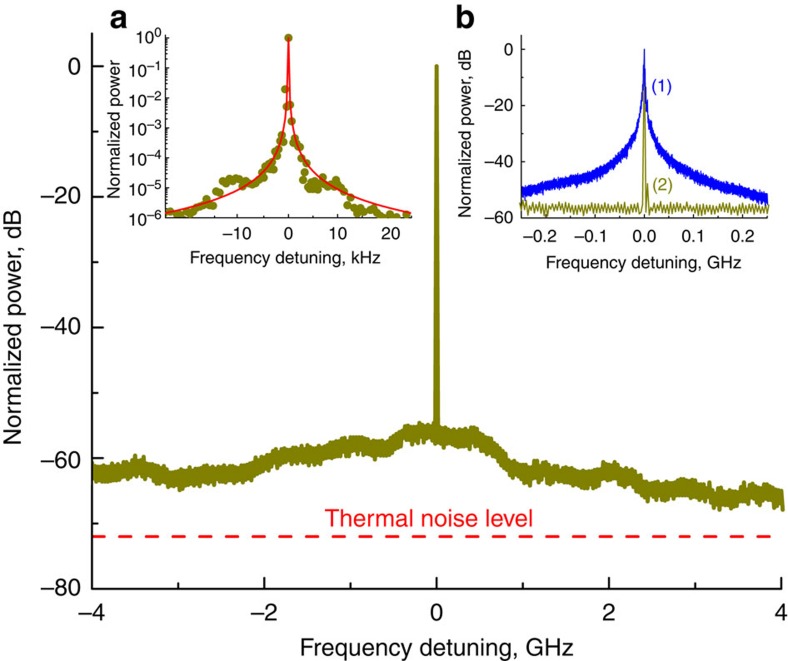
Power spectrum of the RF signal generated by beating two self-injection locked DFB lasers on a fast photodiode. RF carrier frequency is kept at 8.7 GHz, resolution bandwidth is 300 kHz and video bandwidth is 3 kHz. The noise floor is determined by Johnson–Nyquist noise of the photodiode. Inset (**a**): linewidth measurement performed with 30-Hz-resolution bandwidth. Points stand for the experimental data. Continuous red line is a 60-Hz Lorentzian fit of the data. Inset (**b**): comparison of the RF spectra generated by beating of two self-injection locked lasers (curve (2)) and one injection locked and one free running lasers (curve (1)).

The power spectrum of the RF beat note is shown in Fig. 2. Inset (a) of the figure illustrates details of the laser line in the vicinity of the carrier. Inset (b) of the figure compares the spectra produced by beating two self-injection locked lasers (curve (2) in the inset (b) of Fig. 2) as well as a self-injection locked laser and a free running laser (curve (1) in the inset (b) of Fig. 2). The contrast of the measured tone is very good. The noise floor of the spectrum is slightly above the thermal (Johnson) noise limit, which is typical for self-injection locking. The shot noise is smaller than the thermal noise for this particular measurement.

**Figure 3:**
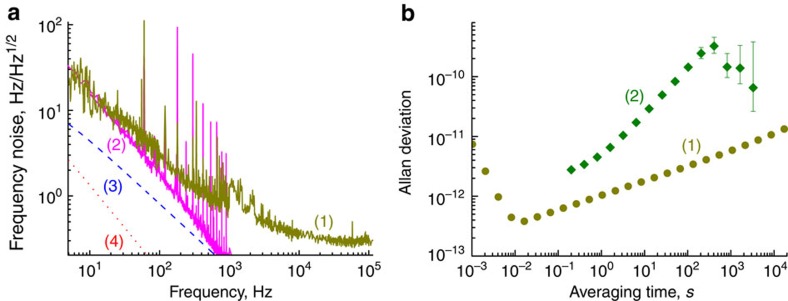
Spectral purity and stability characteristics of the laser. (**a**) Linear frequency noise of the RF beat note of the lasers, (1) compared with the noise determined by conversion of the laser power fluctuations to the frequency fluctuations, (2) as well as fundamental thermorefractive, (3) and thermoexpansive (4) noise. (**b**) Allan deviation interpolated using the frequency noise data (1) and actual measurement of the Allan deviation (2). The 500-s peak in curve (2) results from the air conditioner cycle in the laboratory. This systematic frequency shift exceeds the internal laser noise rather significantly.

The results of frequency noise measurement of the RF signal are presented by line (1) in Fig. 3. The figure shows that the instantaneous linewidth of the beat note, defined as 
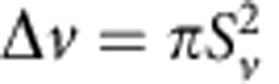
 (here *S*_*ν*_ is the floor of the frequency noise shown in Fig. 3), is less than 0.1 Hz. The free running laser has 2 MHz instantaneous linewidth (see inset in Fig. 3). This demonstrates that self-injection locking improved the DFB laser linewidth by nearly seven orders of magnitude.

The notion of instantaneous linewidth is not strictly applicable to a generic laser suffering from flicker noise. To determine an effective integral linewidth Δ*ν*_eff_ an integral equation is used^47^,^48^


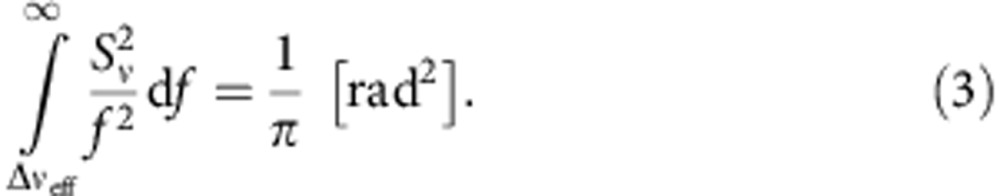


The linewidth found in this way corresponds to equation (1) in the ideal case of an oscillator with white frequency noise. We fitted the frequency noise dependence using decomposition and calculated the integral. The resultant integral linewidth is 50 Hz. Hence, a single laser has less than 30 Hz integral linewidth. The fitted frequency noise was also used to interpolate Allan deviation of the beat note, *σ*(*τ*), using formula


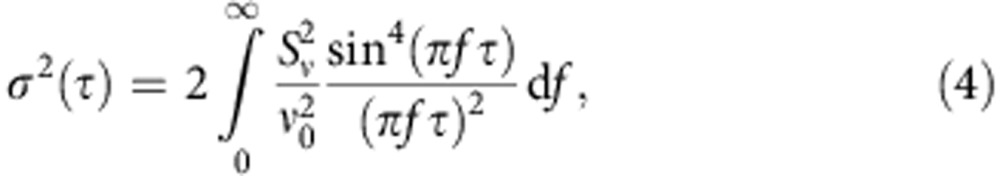


which is shown in the inset of Fig. 3.

To find out which factors limit the performance of the laser, we evaluated the fundamental thermorefractive as well as thermoexpansive frequency noise of the reference WGM resonator^49^. The spectral density of the thermorefractive frequency noise is found from


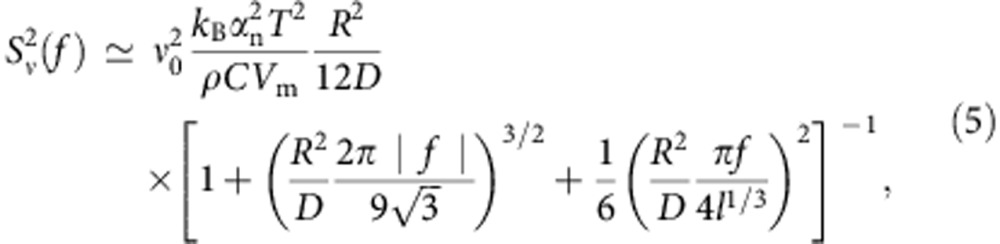


where *R* is the resonator radius, *T* is the ambient temperature, *α*_*n*_ is the thermorefractive coefficient of the material, *V*_m_ is the volume of the WGM mode, *l*=2*πRn*/*λ* is the mode order, *n* is the refractive index of the material, *D* is the temperature diffusion coefficient, *ρ* is the material density and *C* is the specific heat capacity.

To find the impact of thermodynamic fluctuations of the resonator dimension due to conversion of the temperature fluctuations we used the formula


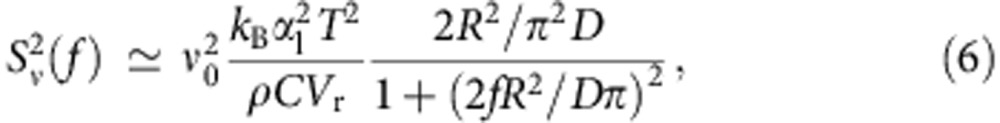


where *α*_l_ is the coefficient of linear thermal expansion of the material and *V*_r_ is the resonator volume.

It is important to find the correct value of the volume of the resonator mode, *V*_m_, to properly use equation (5). Our resonator does not have a morphology that can be readily described analytically. We have performed numerical simulation to analyse the resonator utilized in the experiment using the COMSOL software to solve the corresponding Helmholtz equation. The three-dimensional problem was reduced to a two-dimensional problem because of Azimuthal symmetry of the cavity modes. The field profile of the resonator mode is shown in inset of Fig. 1. The evaluated mode volume is *V*_m_=2.5 × 10^−6 ^cm^3^.

We used MgF_2_ resonators of radius *R*=0.3 cm and thickness 0.01 cm in our experiments. The thermal diffusivity for MgF_2_ is equal to *D*=7.2 × 10^−2 ^cm^2 ^s^−1^ and the characteristic frequency for the thermodynamic processes is *D*/*R*^2^=0.8 s^−1^. The other parameters are *T*=300 K, *α*_*n*_=0.6 × 10^−6 ^K^−1^, *α*_l_=9 × 10^−6 ^K^−1^, *l*=1.7 × 10^4^, *ρ*=3.18 g cm^−3^, *C*=9.2 × 10^6 ^erg g^−1 ^K^−1^, *V*_r_=2.8 × 10^−3 ^cm^3^. The results of the calculations are presented by curves (3) and (4) in Fig. 3. The simulations show that the fundamental limit is ∼10 dB away from the measurement result at small offset frequencies *f*.

## Discussion

To find the reason for the stability limitation we performed two experiments. In one experiment we measured how the frequency of the laser depends on the frequency of the amplitude modulation of the light that enters the resonator. This measurement allowed us to evaluate the transfer function for the laser power noise to the laser frequency. In the other measurement we found the power noise of our laser operating in the self-injection locked regime. Using these results we evaluated the frequency noise due to transfer of the laser amplitude noise to the laser frequency. The result is shown in Fig. 3 by curve (2). In accordance with the measurement we can conclude with high confidence that the laser power (amplitude) noise transfer limits the performance of our laser system. The amplitude noise can be reduced with an amplitude servo that would improve the performance of the laser.

The demonstrated level of frequency stability is not good enough for state-of-the-art optical atomic clocks, which need optical sources with Allan deviation of 10^−15^ or better at 1 s. We expect that the noise performance of the laser can be improved by an order of magnitude via refinement of the technology, reduction of the intracavity optical loss, increase in the mode volume by means of resonator morphology engineering and improvement of the thermal stabilization of the resonator. Another two orders of magnitude improvement can be achieved using self-referencing techniques^50^,^51^,^52^,^53^.

In conclusion, we have demonstrated a heterogeneously integrated chip-scale semiconductor laser with 30 Hz integral and sub-Hz instantaneous linewidths. The laser is completely self-contained in a chip-scale package that includes the semiconductor diode and the reference microresonator. The achieved frequency noise of the laser is on the order of 0.3 Hz Hz^−1/2^ above 10 kHz. The noise can be further improved since the fundamental thermodynamics limits are not reached. The performance of the laser is comparable to the performance of the best bench-top solid state and fibre lasers, while featuring several orders of magnitude smaller volume. Such lasers are particularly important for transition of advanced optical metrology used in applications including atomic clocks, spectroscopy, LIDAR and others, from the laboratory to the field. As an additional feature not available with bench-top laser systems, these miniature lasers have low acceleration sensitivity (relative frequency stability better than 10^−11 ^g^−1^, where g is the free fall acceleration constant, see ref. 54 for a discussion). Thus, they can be used on moving platforms outside the laboratory.

## Additional information

**How to cite this article:** Liang, W. *et al.* Ultralow noise miniature external cavity semiconductor laser. *Nat. Commun.* 6:7371 doi: 10.1038/ncomms8371 (2015).
